# Neuron-Enriched Gene Expression Patterns are Regionally Anti-Correlated with Oligodendrocyte-Enriched Patterns in the Adult Mouse and Human Brain

**DOI:** 10.3389/fnins.2013.00005

**Published:** 2013-02-04

**Authors:** Powell Patrick Cheng Tan, Leon French, Paul Pavlidis

**Affiliations:** ^1^Bioinformatics Graduate Program, University of British ColumbiaVancouver, BC, Canada; ^2^Centre for High-Throughput Biology, University of British ColumbiaVancouver, BC, Canada; ^3^Department of Psychiatry, University of British ColumbiaVancouver, BC, Canada

**Keywords:** gene expression pattern, transcriptome, cell type, neuron, glia, evolution

## Abstract

An important goal in neuroscience is to understand gene expression patterns in the brain. The recent availability of comprehensive and detailed expression atlases for mouse and human creates opportunities to discover global patterns and perform cross-species comparisons. Recently we reported that the major source of variation in gene transcript expression in the adult normal mouse brain can be parsimoniously explained as reflecting regional variation in glia to neuron ratios, and is correlated with degree of connectivity and location in the brain along the anterior-posterior axis. Here we extend this investigation to two gene expression assays of adult normal human brains that consisted of over 300 brain region samples, and perform comparative analyses of brain-wide expression patterns to the mouse. We performed principal components analysis (PCA) on the regional gene expression of the adult human brain to identify the expression pattern that has the largest variance. As in the mouse, we observed that the first principal component is composed of two anti-correlated patterns enriched in oligodendrocyte and neuron markers respectively. However, we also observed interesting discordant patterns between the two species. For example, a few mouse neuron markers show expression patterns that are more correlated with the human oligodendrocyte-enriched pattern and vice-versa. In conclusion, our work provides insights into human brain function and evolution by probing global relationships between regional cell type marker expression patterns in the human and mouse brain.

## 1 Introduction

Gene expression in the adult mammalian brain is highly complex and poorly understood. Over 80% of all genes are expressed in the central nervous system, often with patterns that vary in time and space (Lein et al., 2007; Kang et al., 2011; Hawrylycz et al., 2012). Many genes show patterns that correspond to classical neuroanatomical subdivisions (Lein et al., 2007). Others reflect neurotransmitter systems, and yet others appear to reflect patterns laid down during development (Zapala et al., 2005; Cohen and Greenberg, 2008; Kang et al., 2011). The functional significance of many other patterns is not clear. As the neuroscience community increasingly integrates data across modalities, gaining a deeper understanding of expression patterns is important. One way to gain insight into these patterns is to examine their conservation in evolution. Another is to dissect them into sub-patterns that reflect different cell types. Progress on both of these fronts is enabled by the availability of large-scale data sets. In this paper we focus on expression patterns in the normal adult human brain, comparing them to expression in the normal adult mouse, extending our recent work (French et al., 2011).

There is a broad expectation that gene expression in the mouse and human should be similar, and the brain is no exception. It is well known that the fundamental anatomical structure and function of the nervous system is common across mammals. This is exemplified by the similarities observed in the gene expression patterns in the subcortical regions of the brain (Liao and Zhang, 2006; Strand et al., 2007). Gene expression in the cortical regions on the other hand show greater gene expression diversity between mouse and human (Zeng et al., 2012). Differences in gene expression may be due to the increased number of cortical neurons in primates compared to rodents (Herculano-Houzel, 2009). However, none of these studies is comprehensive in terms of brain regions or genes and insights into studies that look at cell type compositions have been limited. Within specific brain regions, inverse relationships between cell type expression patterns have been observed in human (Oldham et al., 2008). However, it is unclear whether expression patterns are also anti-correlated between brain regions. Recently we reported that gene expression across adult mouse brain regions is dominated by patterns associated with neuron and oligodendrocyte marker expression levels (French et al., 2011). These patterns were identified by seeking strong anti-correlated patterns of gene expression and also by principal component analysis (PCA). PCA captures the dominant patterns in the data in orthogonal variables termed principal components (Pearson, 1901). In the adult mouse brain, higher levels of expression of genes with a neuron-enriched pattern tended to be associated with anterior regions and regions with higher macroconnectivity (French et al., 2011). The opposite was observed for the oligodendrocyte-enriched pattern. We hypothesized that similar relationships exist in the human brain.

To investigate the gene expression patterns in the human brain, we applied PCA to the regional transcriptomes of two adult human brains. Based on the first principal component (PC1) scores, we identified two groups of genes that were enriched for neuron cell type markers (the “neuron-enriched” pattern) and oligodendrocyte cell type markers (the “oligodendrocyte-enriched” pattern) respectively. Our results show that the significant portion of the transcriptome can be explained by the expression of neuron and oligodendrocyte cell type markers which are anti-correlated across brain regions. Moreover, in comparison to mouse subcortical regions, we report homologous genes with similar expression patterns which are also enriched for neuron and oligodendrocyte markers but not astrocyte markers. We also observed homologous genes with differences in expression patterns, the details of these patterns could provide additional insights into functional similarities and differences among mammalian brain lineages.

## 2 Materials and Methods

**Figure 1:**
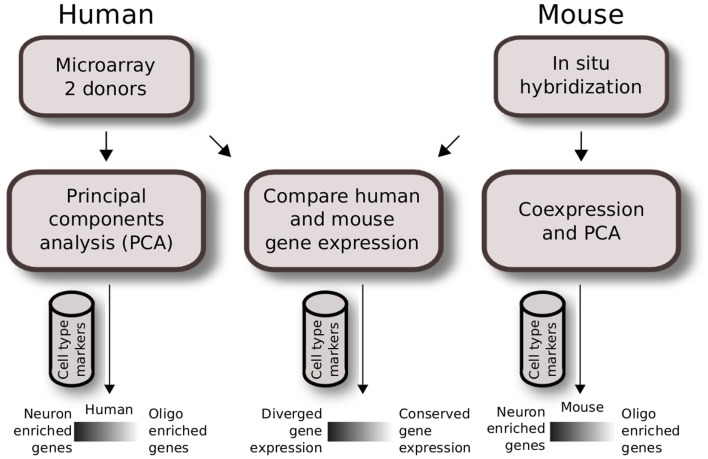
**Analysis workflow of human and mouse gene expression across brain regions**. Quality control for expressing genes and gray matter tissue samples were applied prior to analysis. Regional gene expression patterns were defined using PCA for two human brain microarray data in the first analysis (left). A similar method was applied to mouse ISH data previously as described in French et al. (2011) (right). The second analysis compares homologous data matrices of human H0351.2001 and mouse (middle). Cahoy et al. (2008) cell type markers were used to define neuron and oligodendrocyte-enriched patterns.

We used publicly available datasets and performed two independent analyses to study cell type expression patterns within the human brain and between the mouse and human brain. The overview of the Materials and Methods used are shown in Figure 1.

### 2.1 Human brain gene expression

We analyzed the normalized gene expression data from two healthy adult human post-mortem brains downloaded from the publicly available dataset called the “Allen Human Brain Atlas” provided by the AIBS (Allen Institute for Brain Science; http://www.brain-map.org/; Hawrylycz et al., 2012). Briefly, donor H0351.2001 was a 24-year-old African American male and donor H0351.2002 was a 39-year-old African American male. For both brains, larger regions were manually macrodissected whereas smaller regions were laser captured microdissected. There are 896 brain region samples in the H0351.2001 dataset while the H0351.2002 dataset had 946 samples. The two human datasets were processed and analyzed separately. Sample replicates with the same “structure_name” column annotation were averaged, yielding 323 columns for H0351.2001 and 346 columns for H0351.2002. Samples from the left and right hemispheres were kept separate. Samples of white matter tracts (corpus callosum and cingulum bundle) were excluded from both matrices which resulted in 320 columns in the H0351.2001 dataset and 345 columns in the H0351.2002 dataset. Each normalized gene expression matrix contained data for 58,691 probes. We combined multiple probes for the same gene by taking the mean, yielding expression levels for 29,191 genes.

### 2.2 Mouse brain gene expression

We used the mouse gene expression data from the “Allen Mouse Brain Atlas” as described in our previous study (French and Pavlidis, 2011). Briefly, colorimetric ISH images were collected from adult male, 56-day-old C57BL/6J normal mouse brains (Lein et al., 2007). The ISH images were previously quantified and registered to a 3D reference atlas by Ng et al. (2007). The resulting brain region level expression energy (hereafter referred to as gene expression) is defined as the product of the expression area and the expression intensity (Ng et al., 2009). Missing values are reported as NAs. The resulting mouse expression matrix has 20,444 genes and 207 brain regions.

### 2.3 Human brain analysis

For the analysis of the human data (independent of the mouse data), we focused our analysis on regionally variable gray matter expressed genes by discarding genes with standard deviation or mean expression below the 25th percentile. After filtering, the H0351.2001 dataset had 14,595 genes and 320 brain regions while the H0351.2002 dataset had 14,615 genes and 345 brain regions.

We mean-centered and scaled the expression of each gene by its standard deviation across brain regions by using the “scale” function in R (R Core Team, 2012). The “prcomp” function in R was used to calculate the principal components of the scaled gene expression matrix. PC scores for each gene correspond to the “x” value while PC loadings for each brain region correspond to the “rotation” value of the “prcomp” result. For consistency, we used the convention that the oligodendrocyte marker *MOBP* has a positive PC1 score and adjusted the signs accordingly.

We measured the cell type enrichment in PC1 scores by measuring the area under the curve (AUC) of the receiver operating characteristic curves, in a manner similar to the “wilcox.test” function in R. First, we ranked genes by their PC1 scores. Second, we divided the ranked list of genes into the positive and negative gene sets. This condition depends on the cell type of interest. For example, when we calculated the AUC for neuron markers, those genes that were found in the Cahoy neuron marker list were included in the positive gene set and all other genes were included in the negative gene set. We reversed the sign of the PC1 scores when calculating the AUC for oligodendrocyte markers.

### 2.4 Human-mouse comparisons

Human H0351.2001 and mouse brain region names were manually matched using the sample annotations and ontologies provided by the AIBS. Human genes were converted to mouse genes using HomoloGene build 66 (Wheeler et al., 2007).

We manually compared each brain region name in the AIBS mouse and human structure ontology files. For this analysis, we averaged the gene expression of both left and right human brain hemispheres with a matching structure name. However, there are many brain regions with structure names that do not match between species. To circumvent this, for each species, each brain region was manually annotated with a parent structure that is common to both species. Gene expression of multiple brain regions with the same parent structure were averaged. For example, the human regions “CA1”-“CA4” were averaged to match the parent structure “Ammon’s horn.” Likewise, the mouse regions “Lateral group of the dorsal thalamus,” “Lateral posterior nucleus of the thalamus,” and “Suprageniculate nucleus” were averaged to match the parent structure “Lateral group of Nuclei, Dorsal Division” (Tables S1 and S2 in Supplementary Material).

Gene expression values of both matrices were then quantile normalized. Finally, genes with expression levels below the 25th percentile in both species were removed. The resulting matched human and mouse matrices represent expression values of 7,911 genes across 58 subcortical brain regions.

We calculated the Spearman rank correlation for each homologous gene and quantified cell type marker enrichment in a similar manner to how AUC was calculated from PC1 scores.

### 2.5 Statistical analysis

We used the “cor.test” function in R to calculate Spearman rank correlations together with matching p-values. P-values were corrected for multiple testing by controlling for the false discovery rate, which are reported as q-values (Benjamini and Hochberg, 1995). The distribution of orthologous gene expression pattern correlations was compared to 20 random distributions where human gene labels were shuffled without replacement. Correlations for data with missing values were calculated by using the “pairwise” method of the “cor” function in R (R Core Team, 2012).

Hierarchical clustering was performed with the “hclust” function in R (R Core Team, 2012), using Euclidean distances and Ward’s minimum variance method as parameters (Ward, 1963).

Gene ontology analysis for the 100 most positively and negatively correlated expression patterns were performed using DAVID (Dennis et al., 2003).

### 2.6 Additional data sources

Cell type markers were obtained from Cahoy et al. (2008). Only those marker genes that have at least 10× fold enrichment were used. In H0351.2001, there are a total of 267 neuron, 103 oligodendrocyte, and 143 astrocyte cell type markers that are homologous to the mouse study. Similarly, the H0351.2002 dataset has 270 neuron, 104 oligodendrocyte, and 145 astrocyte markers.

White matter to gray matter (WM/GM) transcript ratios within the anterior cingulate gyrus were obtained from Sibille et al. (2008). Sibille et al. defined WM/GM transcript ratio for each gene in each brain area as the ratio between the average expression of using all samples in the gray matter area and the average expression of using all samples in the adjacent white matter area. Ratios of multiple probe sets for the same gene were averaged. Glia to neuron cell ratios for the human cerebellum, cerebral cortex, and the rest of the brain were obtained from Azevedo et al. (2009) who applied a chemomechanical dissociation technique to purify cells which were labeled by immunohistochemistry.

In relation with mouse and human expression pattern differences, the list of 73 genes that show differential expression pattern between mouse and human visual and temporal cortices was obtained from Zeng et al. (2012). Genes with discordant expression patterns between species were obtained from the list of 49 human-specific markers (genes that are correlated with modules enriched for cell types in human but not in mouse) in the meta-analysis of brain expression performed by Miller et al. (2010). These brain regions include both cortical and subcortical regions.

## 3 Results

### 3.1 Neuron-enriched and oligodendrocyte-enriched patterns are conserved

**Figure 2:**
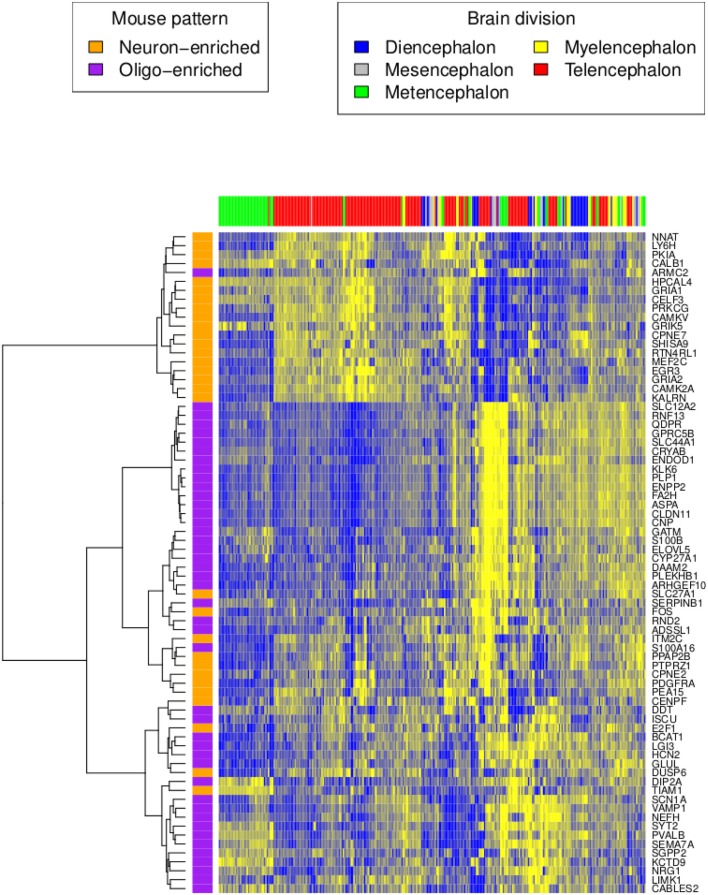
**Human H0351.2001 gene expression profile of orthologous genes reported in the mouse neuron and oligodendrocyte-enriched patterns**. High and low expression levels are colored in yellow and blue respectively. Rows are genes colored by their homolog cell type enrichment. Columns are brain region samples colored by major brain divisions. Hierarchical clustering was performed using the Ward’s minimum variance method in R (Ward, 1963).

We characterized gene expression profiling data from two adult human brains (identified by the Allen Institute for Brain Science (AIBS) as donors H0351.2001 and H0351.2002) in a manner comparable to our previous analysis of the adult mouse brain (Figure 1). After filtering (see Materials and Methods), the H0351.2001 dataset had 14,595 genes while the H0351.2002 dataset had 14,615 genes, 13,250 of which were found in both datasets. For H0351.2001, we obtained 320 brain region samples. Telencephalon accounts for most of the brain region samples (53%), metencephalon (22.1%), diencephalon (11%), myelencephalon (8.1%), and mesencephalon the least (5.9%). The H0351.2002 dataset had 345 samples with similar proportions of major brain divisions as H0351.2001. In H0351.2001, cerebellar samples clustered more closely compared to other brain regions (Figure 2), in line with previous observations that cerebellum gene expression is the most unique compared to other major brain divisions (Sandberg et al., 2000; Lockhart and Barlow, 2001; Pavlidis and Noble, 2001). This was less apparent in H0351.2002 (data not shown). Hereafter, we report results based on these filtered datasets.

Next, we tested whether genes that express anti-correlated cell type enriched patterns in mouse are also anti-correlated in humans (French et al., 2011). We averaged the expression of all human homologs with the mouse neuron-enriched pattern. Similarly, we also averaged the expression pattern of all human homologs with the mouse oligodendrocyte-enriched pattern. As in mouse, the averaged neuron-enriched pattern is anti-correlated with the averaged oligodendrocyte-enriched pattern (H0351.2001 rho = −0.40, P < 0.0001 and H0351.2002 rho = −0.61, P < 0.0001; Figure 2). Genes that show neuron-enriched patterns are predominantly expressed in metencephalon and telencephalon regions while genes in the oligodendrocyte-enriched patterns are not restricted to any major brain division.

This conservation of cell type marker enriched patterns is also evident in a PCA of the human data. The first three principal components of H0351.2001 accounted for 15.6, 11.6, and 8.31% of the total variance respectively whereas we see a slight decrease in the case of H0351.2002 with 15.2, 8.07, and 5.98% of the total variance respectively. The first principal component (PC1) gene scores of the two human datasets are strongly positively correlated (rho = 0.72, P < 0.0001), indicating that overall, the two brains have similar dominant expression patterns, consistent with the findings of Hawrylycz et al. (2012). We observed that these oligodendrocyte and neuron marker genes tend to have PC1 scores with opposite signs, consistent with our previous study in mouse. We term these as “oligodendrocyte-enriched” and “neuron-enriched” respectively. The top 25 genes in the “oligodendrocyte-enriched” and “neuron-enriched” gene sets are shown in Tables 1 and 2 respectively. The complete list of PC1 gene and brain loadings of both donors are available as supplementary material (Tables S3, S4, and S5). For each cell type, we measured the cell type enrichment by comparing the PC1 ranks of those cell type marker genes (as determined by Cahoy) against the PC1 ranks of the remaining genes (see Materials and Methods). In H0351.2001, neuronal markers showed the highest enrichment (AUC = 0.77), followed by oligodendrocyte markers (AUC = 0.73), and astrocyte markers the least (AUC = 0.66; Figure S1 in Supplementary Material). We found evidence for comparable cell type marker enrichment in H0351.2002 PC1 loadings as well (neuron markers AUC = 0.82, oligodendrocyte markers AUC = 0.81, astrocyte markers AUC = 0.63; Figure S2 in Supplementary Material). By way of comparison, in mouse we had found that PC2 gene loadings showed the highest enrichment for oligodendrocyte markers (AUC = 0.77) and neuron markers (AUC = 0.63) and no enrichment for astrocyte markers (AUC = 0.52; Figure S3 in Supplementary Material; French et al., 2011).

**Table 1:** **Top 25 genes in the oligodendrocyte-enriched gene set of human H0351.2001 sorted by PC1 score**.

Gene symbol	Gene description	Entrez ID	PC1	Mean	SD
SLC27A1	Solute carrier family 27 (fatty acid transporter), member 1	376497	12.67	10.92	0.33
REST	RE1-silencing transcription factor	5978	12.46	4.65	0.43
PPARA	Peroxisome proliferator-activated receptor alpha	5465	12.41	3.65	0.37
ARHGEF10	Rho guanine nucleotide exchange factor (GEF) 10	9639	12.33	5.19	0.42
TRIM56	Tripartite motif-containing 56	81844	12.14	3.93	0.42
EGFR	Epidermal growth factor receptor	1956	12.12	4.21	0.55
A_24_P943258	AGILENT probe A_24_P943258 (non-RefSeq)	NA	12.11	4.92	0.43
A_23_P129258	AGILENT probe A_23_P129258 (non-RefSeq)	NA	12.01	13.75	0.51
RBMS2	RNA binding motif, single stranded interacting protein 2	5939	12.00	6.32	0.39
A_24_P316059	AGILENT probe A_24_P316059 (non-RefSeq)	NA	11.98	4.83	0.39
GPR75	G protein-coupled receptor 75	10936	11.96	6.83	0.39
PFKFB3	6-Phosphofructo-2-kinase/fructose-2,6-biphosphatase 3	5209	11.95	7.15	0.39
C12orf39	Chromosome 12 open reading frame 39	80763	11.95	4.65	0.47
MAFIP	MAFF interacting protein	727764	11.84	5.13	0.36
CXorf36	Chromosome X open reading frame 36	79742	11.82	2.58	0.42
NPAS3	Neuronal PAS domain protein 3	64067	11.75	8.08	0.35
SDPR	Serum deprivation response	8436	11.74	3.43	0.44
LIMS1	LIM and senescent cell antigen-like domains 1	3987	11.74	3.45	0.35
A_24_P475689	AGILENT probe A_24_P475689 (non-RefSeq)	NA	11.67	3.67	0.43
BMP7	Bone morphogenetic protein 7	655	11.59	7.21	0.38
CTNNA1	Catenin (cadherin-associated protein), alpha 1, 102 kDa	1495	11.58	6.69	0.41
TJP1	Tight junction protein 1 (zona occludens 1)	7082	11.53	8.61	0.34
KIF19	Kinesin family member 19	124602	11.53	3.02	0.50
A_23_P134887	AGILENT probe A_23_P134887 (non-RefSeq)	NA	11.53	5.72	0.49
F11	Coagulation factor XI	2160	11.50	3.10	0.41

**Table 2:** **Top 25 genes in the neuron-enriched gene set of human H0351.2001 sorted by PC1 score**.

Gene symbol	Gene description	Entrez ID	PC1	Mean	SD
RNF41	Ring finger protein 41	10193	−18.90	6.48	0.39
ARF5	ADP-ribosylation factor 5	381	−18.87	7.80	0.47
A_32_P86533	AGILENT probe A_32_P86533 (non-RefSeq)	NA	−18.49	8.19	0.64
GSTA4	Glutathione S-transferase alpha 4	2941	−18.49	7.76	0.34
MMS19	MMS19 nucleotide excision repair homolog	64210	−18.26	6.51	0.40
TMEM59L	Transmembrane protein 59-like	25789	−18.26	6.55	0.71
CLTA	Clathrin, light chain A	1211	−18.26	9.45	0.38
UBE2K	Ubiquitin-conjugating enzyme E2K	3093	−18.24	9.58	0.36
AP2A2	Adaptor-related protein complex 2, alpha-2 subunit	161	−18.22	8.50	0.36
NMNAT2	Nicotinamide nucleotide adenylyltransferase 2	23057	−18.11	8.75	0.53
LCMT1	Leucine carboxyl methyltransferase 1	51451	−18.07	8.87	0.34
PDCD2L	Programmed cell death 2-like	84306	−17.99	6.54	0.41
LOC727967	Similar to block of proliferation 1	727967	−17.92	6.31	0.44
HAGH	Hydroxyacylglutathione hydrolase	3029	−17.89	8.14	0.50
DHX30	DEAH (Asp-Glu-Ala-His) box polypeptide 30	22907	−17.88	6.94	0.42
RTN1	Reticulon 1	6252	−17.87	9.77	0.61
CCT2	Chaperonin containing TCP1, subunit 2 (beta)	10576	−17.81	10.38	0.37
PI4KA	Phosphatidylinositol 4-kinase, catalytic, alpha	5297	−17.80	8.38	0.41
IARS	Isoleucyl-tRNA synthetase	3376	−17.78	7.50	0.38
ABHD14A	Abhydrolase domain containing 14A	25864	−17.76	7.74	0.42
PLD3	Phospholipase D family, member 3	23646	−17.76	8.49	0.61
ATP6AP1	ATPase, H+ transporting, lysosomal accessory protein 1	537	−17.74	8.99	0.45
C19orf62	Chromosome 19 open reading frame 62	29086	−17.66	7.36	0.43
RAB24	RAB24, member RAS oncogene family	53917	−17.64	8.06	0.38
KLHDC3	Kelch domain containing 3	116138	−17.63	6.60	0.43

### 3.2 Principal component loadings partly reflect varying cell type proportions

The PC1 gene loadings could either be explained by variations in expression levels within cells, or by variations in the ratio of different sub-populations of cells (or some combination of these). To further investigate this, we calculated the correlation between the H0351.2001 PC1 gene loadings and the white matter to gray matter transcript ratio (WM/GM) for 8,088 genes with data for both (Sibille et al., 2008). The correlation is statistically significant (rho = 0.59, P < 0.0001). Since white matter regions have been excluded from the human data we used, we interpret the WM/GM transcript ratios as variations in cell type proportions within gray matter regions.

**Figure 3:**
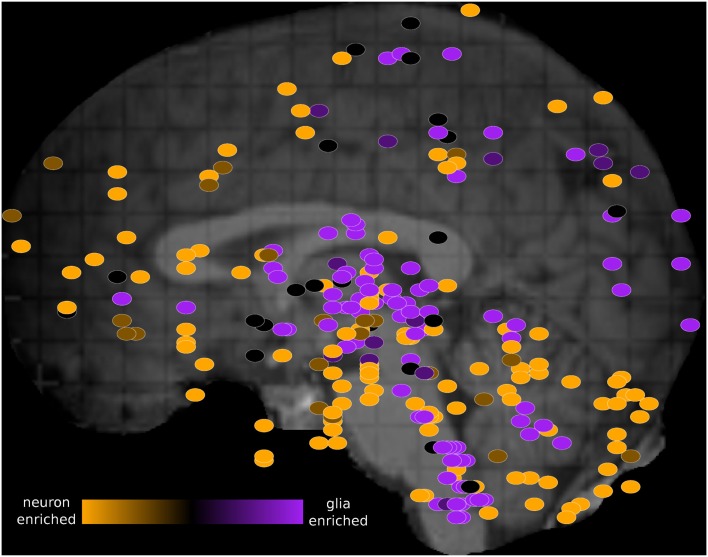
**Schematic view of the H0351.2001 human brain showing oligodendrocyte-neuron PC1 marker ratio within each brain region sample**. The brain PC1 loadings were obtained from the rotation attribute result object of the “prcomp” function in R. PC1 brain loadings range from 0.03 (orange) to −0.04 (purple) which suggest increasing glia-neuron ratio. Primary and secondary axes correspond to the mri_z and mri_y coordinates respectively. These dots were manually overlaid onto a brain image from the Allen Brain Explorer 2 software (http://mouse.brain-map.org/static/brainexplorer). In order to visualize subcortical region samples, we have hidden the visualization of the left cerebral hemisphere which causes some cortical samples (such as part of the left temporal cortex) to appear outside of the brain.

We visualized the PC1 loadings on the schematic image of the brain using the Allen Brain Explorer 2 (see Materials and Methods; Figure 3 and Table S4 in Supplementary Material). Regions where there is high neuron marker expression include inferior frontal gyrus, CA2, and temporal pole. Regions where there is high oligodendrocyte marker expression include globus pallidus, putamen, and head of caudate nucleus, in agreement with the enrichment of these regions in myelinated axons (Feher, 2012).

In addition, we calculated the ratio between “oligodendrocyte-enriched” PC1 markers and “neuron-enriched PC1” markers and compared it to the glia to neuron ratio measurements performed by Azevedo et al. (2009). In agreement, in H0351.2001, we find that the human cerebellum, cerebral gray matter, and the rest of the brain samples show increasing glia to neuron ratio respectively (Table 3). In H0351.2002, the cerebellum shows higher glia to neuron ratio than cerebral gray matter which may be due to individual variability or technical artifacts.

**Table 3:** **PC1 brain loadings (mean ± standard deviation) of the two AIBS human datasets and measured glia to neuron ratio from Azevedo et al. (2009) in cerebellum, cerebral gray matter, and remaining brain regions**.

Brain division	H0351.2001	H0351.2002	Azevedo et al. (2009)
Cerebellum	−0.032 ± 0.062	0.013 ± 0.045	0.23
Cerebral gray matter	−0.00069 ± 0.058	−0.012 ± 0.059	1.48
Rest of the brain	0.016 ± 0.042	0.015 ± 0.042	11.35

Together, these results suggest that gene expression variance in the human brain can partly be explained by variations in cell type composition, though we cannot exclude contributions from changes in expression within cell types.

### 3.3 Orthologous genes with positively correlated expression patterns are enriched in cell type markers

In addition to identifying dominant gene expression patterns within each species, we also performed a comparison of gene expression patterns between orthologous gene and brain region samples in mouse and in human AIBS data, focusing on the H0351.2001 dataset which we deem to be the higher quality of the two data sets based on the clustering of cerebellar regions described above. Within data sets, we found that regional expression patterns show greater homogeneity in human (mean Spearman rho = 0.98 ± 0.0079) than in mouse (mean Spearman rho = 0.86 ± 0.022). That is, expression patterns across mouse brain regions were apparently more variable than across human brain regions, possibly for technical reasons. Next, we measured the conservation of gene expression patterns by measuring the correlation for each homologous gene across matched brain regions. Finally, we compared our results with those of other studies by performing enrichment analyses on genes ranked by the strength of their correlation between species.

To prepare gene expression matrices of the same size, we limited the analysis to genes expressed above the 25th percentile in both species and brain regions which could be matched between mouse and human, resulting in 7,911 genes and 58 subcortical brain regions (see Materials and Methods). Major brain regions include the hippocampal formation, cerebral nuclei, thalamus, epithalamus, hypothalamus, midbrain regions, pons, medulla, and cerebellum. In this filtered data set, we saw consistent cell type marker enrichment in the PC scores in both mouse and human which indicates that the filtering process did not have a large effect on the data with respect to the patterns described in the previous section (data not shown).

We calculated the Spearman rank correlation between pairs of homologous brain regions and found statistically significant positive correlations (mean Spearman rho = 0.31 ± 0.031, P < 0.0001). The three most similar brain regions include Ammon’s horn (rho = 0.40), dentate gyrus (rho = 0.38), and subiculum (rho = 0.35). Brain regions with the poorest correlation include nucleus raphe pontis (rho = 0.21), gracile nucleus (rho = 0.25), and pallidum (rho = 0.25).

**Figure 4:**
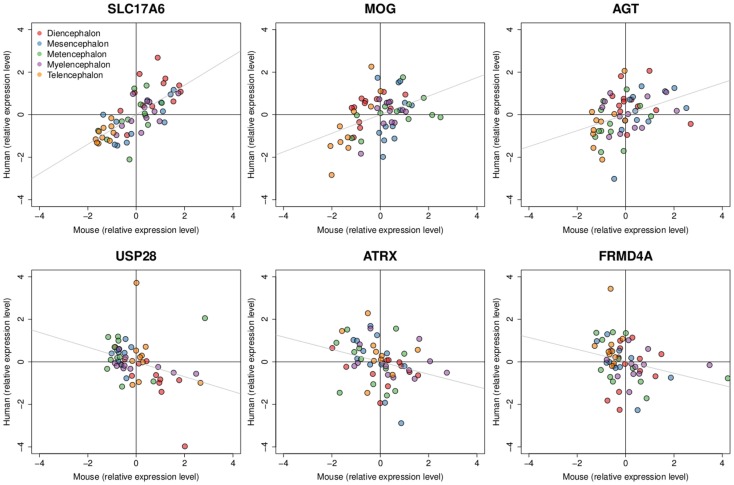
**Examples of positively and negatively correlated gene expression patterns between mouse and human H0351.2001**. Dots represent brain region samples colored by major brain divisions. Three genes with expression patterns that are positively correlated are shown at the top (*SLC17A6* rho = 0.73, *MOG* rho = 0.39, *AGT* rho = 0.44) while negatively correlated gene expression patterns are shown at the bottom (*USP28* rho = −0.44, *ATRX* rho = −0.36, *FRMD4A* rho = −0.36). All six genes have q-values < 0.3. Expression levels are scaled and centered at zero for visualization.

In terms of genes, we measured the Spearman rank correlation of each homologous gene’s expression levels across matched brain regions. We used these correlation values to rank homologous genes, such that those genes with conserved expression patterns are positively correlated while genes with discordant patterns have either no correlation or are anti-correlated across matched regions. When the correlation distribution is plotted, we observed a positive skew in the distribution (mean rho = 0.074, min rho = −0.57, max rho = 0.73; Figure S4 in Supplementary Material). To verify whether this skew is significant or not, we compared this correlation distribution with a random distribution obtained by shuffling gene labels (see Materials and Methods). There are 53 fewer genes with correlation below −0.30 when compared to random while there are 645 more genes with correlation above 0.30 when compared to random. Together, this indicates that there are more genes with similar expression patterns than not. The top 25 genes with the most positively and negatively correlated gene expression between mouse and human are shown in Tables 4 and 5 respectively. Figure 4 shows examples of genes with positively and negatively correlated expression levels across brain regions. We note that when only a few (∼10) especially highly correlated brain regions were selected, the distribution became more positively skewed (data not shown), suggesting that more focused comparisons might provide higher resolution results, but it was not obvious how to choose such regions *a priori*.

**Table 4:** **Top 25 genes with similar expression patterns between mouse (Mouse expression) and human H0351.2001 (Human expression) sorted by Spearman rank correlation (rho) with q < 0.01**.

Gene symbol	Gene description	Rho	Mouse expression	Human expression
KCNC1	Potassium voltage-gated channel, Shaw-related subfamily, member 1	0.73	9.93	3.84
SLC17A6	Solute carrier family 17 (sodium-dependent inorganic phosphate cotransporter), member 6	0.73	7.62	4.65
ZIC1	Zic family member 1 (odd-paired homolog, Drosophila)	0.69	3.71	5.61
PCP4	Purkinje cell protein 4	0.66	8.16	4.69
GABBR2	Gamma-aminobutyric acid (GABA) B receptor, 2	0.66	13.81	4.38
CACNA1C	Calcium channel, voltage-dependent, L type, alpha 1C subunit	0.65	2.63	1.81
CAMK2D	Calcium/calmodulin-dependent protein kinase II delta	0.63	12.37	3.42
OSBPL5	Oxysterol binding protein-like 5	0.63	3.17	2.67
VAT1	Vesicle amine transport protein 1 homolog (*T. californica*)	0.62	1.76	1.73
SLC8A1	Solute carrier family 8 (sodium/calcium exchanger), member 1	0.62	7.77	3.70
PLCB4	Phospholipase C, beta 4	0.61	7.81	4.30
FOXP2	Forkhead box P2	0.61	1.90	1.97
SPOCK1	Sparc/osteonectin, cwcv, and kazal-like domains proteoglycan (testican) 1	0.61	12.2	3.36
C20orf103	Chromosome 20 open reading frame 103	0.61	3.47	3.38
KCNQ3	Potassium voltage-gated channel, KQT-like subfamily, member 3	0.61	4.16	3.82
GNG4	Guanine nucleotide binding protein (G protein), gamma 4	0.60	0.19	0.24
HTR1A	5-Hydroxytryptamine (serotonin) receptor 1A	0.60	1.27	0.85
ZMAT4	Zinc finger, matrin type 4	0.60	2.62	2.80
ADAM11	ADAM metallopeptidase domain 11	0.60	10.30	3.41
NRN1	Neuritin 1	0.60	9.97	5.40
NTNG1	Netrin G1	0.59	6.73	6.15
ST8SIA5	ST8 alpha-N-acetyl-neuraminide alpha-2,8-sialyltransferase 5	0.59	5.81	3.90
HCN1	Hyperpolarization activated cyclic nucleotide-gated potassium channel 1	0.59	2.62	2.39
PCDH11X	Protocadherin 11 X-linked	0.59	0.93	0.62
SYN2	Synapsin II	0.59	10.29	4.76

*Expr corresponds to the mean expression level across brain regions*.

**Table 5:** **Top 25 genes with anti-correlated expression patterns between mouse (Mouse expression) and human H0351.2001 (Human expression) sorted by Spearman rank correlation (rho)**.

Gene symbol	Gene description	Rho	q-Value	Mouse expression	Human expression
TMEM2	Transmembrane protein 2	−0.57	0.001	0.28	0.95
ABCA8	ATP-binding cassette, subfamily A (ABC1), member 8	−0.45	0.020	0.40	0.50
RPIA	Ribose 5-phosphate isomerase A	−0.44	0.026	0.26	0.57
USP28	Ubiquitin specific peptidase 28	−0.44	0.029	2.26	1.55
WARS2	Tryptophanyl tRNA synthetase 2, mitochondrial	−0.43	0.030	0.46	0.38
SMOC2	SPARC related modular calcium binding 2	−0.42	0.037	0.58	1.16
CROT	Carnitine O-octanoyltransferase	−0.41	0.039	2.20	1.54
KIAA1279	KIAA1279	−0.41	0.044	7.40	3.66
ACOX2	Acyl-CoA oxidase 2, branched chain	−0.40	0.068	0.49	0.42
AGGF1	Angiogenic factor with G patch and FHA domains 1	−0.39	0.079	0.28	0.11
MRPS34	Mitochondrial ribosomal protein S34	−0.39	0.062	2.17	1.20
TMLHE	Trimethyllysine hydroxylase, epsilon	−0.39	0.057	2.62	1.44
CTR9	Ctr9, Paf1/RNA polymerase II complex component, homolog	−0.38	0.072	2.86	1.60
TNFRSF11B	Tumor necrosis factor receptor superfamily, member 11b	−0.38	0.097	0.25	0.60
C2orf29	Chromosome 2 open reading frame 29	−0.38	0.073	1.40	0.75
NCF4	Neutrophil cytosolic factor 4, 40 kDa	−0.38	0.078	0.21	0.20
PAICS	Phosphoribosylaminoimidazole carboxylase	−0.38	0.075	1.02	0.94
CEP164	Centrosomal protein 164 kDa	−0.37	0.078	0.91	0.61
CECR5	Cat eye syndrome chromosome region, candidate 5	−0.37	0.098	2.40	1.02
KIAA0174	KIAA0174	−0.36	0.100	4.34	2.09
ATRX	Alpha thalassemia/mental retardation syndrome X-linked	−0.36	0.090	6.84	1.85
DNAJC5	DNAj (Hsp40) homolog, subfamily C, member 5	−0.36	0.097	15.11	3.70
FRMD4A	FERM domain containing 4A	−0.36	0.097	5.73	1.89
RAP1GAP	RAP1 GTPase activating protein	−0.36	0.092	14.17	3.32
CDK4	Cyclin-dependent kinase 4	−0.36	0.590	1.35	1.23

*Expr corresponds to the mean expression level across brain regions*.

Since we observed cell type marker enrichment in the gene expression patterns for each species independently, we hypothesize that homologous genes that show conserved expression patterns are also enriched for cell type markers. We measured the cell type marker enrichment using the ranked list of homologous genes, annotated by cell type in Cahoy et al. (2008) (see Materials and Methods). In line with our hypothesis, our results show that expression patterns of homologous genes are enriched for neuronal (AUC = 0.74) and oligodendrocyte (AUC = 0.71) markers, but not astrocyte (AUC = 0.53) markers (Figure S5 in Supplementary Material; see Materials and Methods). We interpret this as suggesting that neuronal markers and oligodendrocyte markers are generally more conserved in expression patterns in comparison to the non-cell type markers (Table 4), consistent with the findings of Miller et al. (2010). In contrast, astrocyte markers were relatively poorly conserved overall, some astrocyte markers show positively correlated patterns (e.g., *AGT*, *GFAP*) while others show negatively correlated patterns (e.g., *SLC27A1*, *SCARA3*) between mouse and human (Tables 4 and 5). We found similar results when using less stringent criterion for selecting genes from the Cahoy data (at least 5× enrichment instead of 10×, data not shown). We performed a Gene Ontology (GO) enrichment analysis for the top 100 genes with the most positively and negatively correlated patterns. Those genes with similar expression patterns are significantly enriched for Gene Ontology (GO) biological processes such as ion transport (GO:0006811), transmission of nerve impulse (GO:0019226), and synaptic transmission (GO:0007268; Table S6 in Supplementary Material). The top 100 genes with the most negatively correlated patterns are enriched in biological processes such as negative regulation of homeostatic process (GO:0032845), fatty acid oxidation (GO:0019395), and macromolecule catabolic process (GO:0009057).

Discordant expression patterns between mouse and human orthologs might indicate interesting functional divergences. We identified only 78 genes with reasonably strong negative correlations between mouse and human (rho < −0.3). To seek supporting evidence for these and other negative correlations, we compared our findings to two previous mouse-human comparisons. Zeng et al. (2012) identified 73 genes with patterns considered discordant in the neocortex, including differences in laminar distribution. Of these, 12 are negatively correlated in our study, including one of the 78 meeting a threshold of −0.3 (*SLC6A12* rho = −0.31; Table S7 in Supplementary Material). Miller and colleagues identified 49 “human-specific” cell type markers using meta-analysis of microarray data, of which fourteen are negatively correlated in our analysis, of which two are below −0.3 (*KIAA0174* rho = −0.36 and *ADK* rho = −0.32; Table S8 in Supplementary Material). Thus despite major differences in methodology and brain regions considered, some previous reports of mouse-human differences are supported by our analysis.

## 4 Discussion

We studied the dominant gene expression patterns across the human brain and observed similar complementarity between “neuron/oligodendrocyte” enriched patterns as we previously identified in the mouse (French et al., 2011). Our analysis also shows that *in situ* data from mouse can be meaningfully compared to microarray data from human. As Lee et al. (2008) pointed out, comparisons between ISH and microarray data are challenging due to technical differences such as probe sequence sensitivity and specificity, dynamic range normalization method, and mapping of ISH data. Despite these technical differences, we report gene expression pattern similarities as exemplified by the anti-correlation between neuron and oligodendrocyte-enriched patterns. Our interpretation of the cell type enriched pattern in human is similar to our previous interpretation in mouse (French et al., 2011). A simple explanation is that neurons and glia vary in inverse proportions across brain regions in both human and mouse, which shows an anterior-posterior gradient (Figure 3). However, it is difficult to fully verify this because we currently have limited information on the details of the size and proportions of cell types within each brain region sampled.

The strength of the cell type marker enrichment suggests that many other genes, while not reported as cell type markers by Cahoy et al. (2008), are likely to be expressed in a cell type enriched manner. Genes in this category include ones we predict based on our readings to be expressed in neurons such as neural epidermal growth factor-like 2 (*NELL2*), reticulon 4 receptor (*RTN4R*), potassium channel, subfamily K, member 1 (*KCNK1*), and glutaminase (*GLS*) as well as ones we predict to be expressed in oligodendrocytes such as chloride intracellular channel 4 (*CLIC4*), crystallin, alpha B (*CRYAB*), prostaglandin D2 synthase 21 kDa (*PTGDS*), quinoid dihydropteridine reductase (*QDPR*), and G protein-coupled receptor, family C, group 5, member B (*GPRC5B*). Using a literature review, we have confirmed some of these, suggesting their absence from the lists given by Cahoy et al. to be due to technical factors or the choice of cells used in their study. For example, ISH of the adult mouse and rat brains show *RTN4R* (reticulon 4 receptor or Nogo receptor) is strongly expressed within neurons of the neocortex, hippocampal formation, and granule cells of the cerebellum (Hunt et al., 2002). On the other hand, it is also apparent that what we term the “oligodendrocyte-enriched” and “neuron-enriched” patterns are not purely populated by genes specific for those cell types. For example in the H0351.2001 dataset, *TMEM163*, *CNTN1*, and *TMEM2* are Cahoy oligodendrocyte marker genes but are found close to neuronal markers in our PCA, while the converse is true for the neuronal markers *ST8SIA2* and *GPR12*. This complexity presumably in part reflects sub-populations of neurons which have a different physical or regulatory relationship to glial cells than those which occur in the “neuron-enriched” pattern, or vice-versa.

A second goal of our study was to identify similarities and differences in expression pattern between mouse and human brains. Our overall conclusion is that the similarities vastly outnumber the differences. We found that the similarities are most striking for genes which are known to be enriched in neurons and oligodendrocytes (Table 4 and Figure 4). In contrast, markers of astrocytes demonstrate more differences between mouse and human. In mouse, astrocyte markers were equally represented in both “neuron-enriched” and “oligodendrocyte-enriched” patterns (Figure S3 in Supplementary Material; French et al., 2011). In contrast, in the human data, astrocyte markers coordinately vary in expression levels considerably across regions (Figures S1, S2 and S6 in Supplementary Material). Astrocytes support the metabolically demanding tasks of neurons by recycling neurotransmitters and maintaining ion homeostasis in the brain (Blanger et al., 2011). The enrichment seen in humans could be caused by the increased complexity found only in human astrocytes (Oberheim et al., 2009) or by the higher astrocyte to neuron ratio observed with increasing brain complexity (Nedergaard et al., 2003). Aside from astrocyte markers, we found evidence for other genes showing discordant expression patterns. For example, the mouse *ATRX* (alpha thalassemia/mental retardation syndrome X-linked) expression pattern is negatively correlated with human (rho = −0.36, q = 0.09; Figure 4 and Table 5). In adult mouse, this gene has a higher expression in the medulla compared to the amygdala, while the opposite is true in human. We caution that from the available data it is difficult to determine which of the differences we observe reflect true biological differences (e.g., different species isoforms), and which are due to differences between ISH and microarray. However, the partial overlap of our negative correlations with previous reports of mouse-human differences (Miller et al., 2010; Zeng et al., 2012) suggests that some the other differences we report are worthy of further study.

In summary, using PCA, we provide a candidate list of cell type markers which could be useful for targeting specific cell types or specific regional patterns of interest. In addition, we report correlations for the regional expression of genes between mouse and human which can be useful in the development of mouse disease models or in the study of the molecular evolution of the brain. Future studies that explore the different regulatory mechanisms of genes with discordant expression patterns might provide insights into the evolution of brain structure and function. Furthermore, future high-resolution large-scale studies that examine gene expression in developing mouse and human brain will uncover genes that are only active in early development and thus provide a better understanding of human brain evolution.

## 5 Glossary

**AIBS**: Allen Institute for Brain Science (http://www.brain-map.org/) is a non-profit organization that makes publicly available large-scale data that pertains to neuroscience which includes *in situ* images of the mouse brain and human brain microarray.

**Oligodendrocyte-enriched pattern**: a set of genes whose expression levels follows a pattern similar to those of oligodendrocyte markers such as the myelin basic protein gene.

**Neuron-enriched pattern**: a set of genes whose expression levels follows a pattern similar to those of neuron markers such as the neurofilament, light polypeptide gene.

**PCA**: principal component analysis is a statistical technique that projects high dimensional data to lower dimensions in terms of orthogonal variables termed principal components (Pearson, 1901).

## Author Contributions

Powell Patrick Cheng Tan designed and performed the experiments, analyzed data, and wrote the paper; Leon French contributed to the data preprocessing, analysis, and interpretation of results; Paul Pavlidis supervised and was involved throughout the project. All authors discussed the results and implications and commented on the manuscript at all stages.

## Conflict of Interest Statement

The authors declare that the research was conducted in the absence of any commercial or financial relationships that could be construed as a potential conflict of interest.

## Supplementary Material

The Supplementary Material for this article can be found online at http://www.chibi.ubc.ca/NEOE

Supplementary Table S1 **Orthologous human brain annotations**.

Supplementary Table S2 **Orthologous mouse brain annotations**.

Supplementary Table S3 **H0351.2002 PC1 gene loadings**.

Supplementary Table S4 **H0351.2001 brain loadings**.

Supplementary Table S5 **H0351.2002 brain loadings**.

Supplementary Table S6 **Gene set enrichment of 100 genes with the most positive homologous gene correlations**.

Supplementary Table S7 **Gene-gene correlation of genes that show differential expression patterns between species in Zeng et al. (2012)**.

Supplementary Table S8 **Gene-gene correlation of genes that show differential expression patterns between species in Miller et al. (2010)**.

Supplementary Figure S1 **H0351.2001 cell type marker enrichment ROC curves**.

Supplementary Figure S2 **H0351.2002 cell type marker enrichment ROC curves**.

Supplementary Figure S3 **Mouse cell type marker enrichment ROC curves**.

Supplementary Figure S4 **Correlation distribution between homologous genes that are expressed**. Correlation distribution is skewed toward the positive compared to random where human gene labels were shuffled without replacement. The mean correlation is 0.074.

Supplementary Figure S5 **Gene-gene correlation cell type marker enrichment ROC curves**. Expression data were mean-centered scaled.

Supplementary Figure S6 **Relative expression levels of homologous astrocyte markers across brain regions**.

## Abbreviations

AIBS, Allen Institute for Brain Science; AUC, area under curve; GO, gene ontology; H0351.2001, Allen human brain atlas donor profile of a 24-year-old African American male; H0351.2002, Allen human brain atlas donor profile of a 39-year-old African American male; ISH, *In situ* hybridization; PCA, principal component analysis; PC1, First principal component; WM/GM, white matter to gray matter transcript ratio.

## References

[B1] Azevedo F. A. C. Carvalho L. R. B. Grinberg L. T. Farfel J. M. Ferretti R. E. L. Leite R. E. P. (2009). Equal numbers of neuronal and non-neuronal cells make the human brain an isometrically scaled-up primate brain. J. Comp. Neurol. 513, 532–541 10.1002/cne.21974 19226510

[B2] Benjamini Y. Hochberg Y. (1995). Controlling the false discovery rate: a practical and powerful approach to multiple testing. J. R. Stat. Soc. Series B Methodol. 57, 289–300

[B3] Blanger M. Allaman I. Magistretti P. J. (2011). Brain energy metabolism: focus on astrocyte-neuron metabolic cooperation. Cell Metab. 14, 724–738 10.1016/j.cmet.2011.08.016 22152301

[B4] Cahoy J. D. Emery B. Kaushal A. Foo L. C. Zamanian J. L. Christopherson K. S. (2008). A transcriptome database for astrocytes, neurons, and oligodendrocytes: a new resource for understanding brain development and function. J. Neurosci. 28, 264–278 10.1523/JNEUROSCI.4178-07.2008 18171944 PMC6671143

[B5] Cohen S. Greenberg M. E. (2008). Communication between the synapse and the nucleus in neuronal development, plasticity, and disease. Annu. Rev. Cell Dev. Biol. 24, 183–209 10.1146/annurev.cellbio.24.110707.175235 18616423 PMC2709812

[B6] Dennis G. Jr. Sherman B. T. Hosack D. A. Yang J. Gao W. Lane H. C. (2003). DAVID: database for annotation, visualization, and integrated discovery. Genome Biol. 4, P3 10.1186/gb-2003-4-9-r60 12734009 PMC193660

[B7] Feher J. (2012). *Quantitative Human Physiology: An Introduction* Academic Press series in biomedical engineering. Elsevier Science and Technology, ISBN 9780123821638, 0123821649. URL http://textbooks.elsevier.com/web/productdetails.aspx?isbn=9780123821638

[B8] French L. Pavlidis P. (2011). Relationships between gene expression and brain wiring in the adult rodent brain. PLoS Comput. Biol. 7:e1001049 10.1371/journal.pcbi.1001049 21253556 PMC3017102

[B9] French L. Tan P. P. C. Pavlidis P. (2011). Large-scale analysis of gene expression and connectivity in the rodent brain: insights through data integration. Front. Neuroinform. 5:12 10.3389/fninf.2011.00012 21863139 PMC3149147

[B10] Hawrylycz M. J. Lein E. S. Guillozet-Bongaarts A. L. Shen E. H. Ng L. Miller J. A. (2012). An anatomically comprehensive atlas of the adult human brain transcriptome. Nature 489, 391–399 10.1038/nature11405 22996553 PMC4243026

[B11] Herculano-Houzel S. (2009). The human brain in numbers: a linearly scaled-up primate brain. Front. Hum. Neurosci. 3:31 10.3389/neuro.09.031.2009 19915731 PMC2776484

[B12] Hunt D. Mason M. R. J. Campbell G. Coffin R. Anderson P. N. (2002). Nogo receptor mRNA expression in intact and regenerating CNS neurons. Mol. Cell. Neurosci. 20, 537–552 10.1006/mcne.2002.1153 12213438

[B13] Kang H. J. Kawasawa Y. I. Cheng F. Zhu Y. Xu X. Li M. (2011). Spatio-temporal transcriptome of the human brain. Nature 478, 483–489 10.1038/nature10523 22031440 PMC3566780

[B14] Lee C.-K. Sunkin S. M. Kuan C. Thompson C. L. Pathak S. Ng L. (2008). Quantitative methods for genome-scale analysis of in situ hybridization and correlation with microarray data. Genome Biol. 9, R23 10.1186/gb-2008-9-1-r23 18234097 PMC2395252

[B15] Lein E. S. Hawrylycz M. J. Ao N. Ayres M. Bensinger A. Bernard A. (2007). Genome-wide atlas of gene expression in the adult mouse brain. Nature 445, 168–176 10.1038/nature05453 17151600

[B16] Liao B.-Y. Zhang J. (2006). Evolutionary conservation of expression profiles between human and mouse orthologous genes. Mol. Biol. Evol. 23, 530–540 10.1093/molbev/msj119 16280543

[B17] Lockhart D. J. Barlow C. (2001). Expressing what’s on your mind: DNA arrays and the brain. Nat. Rev. Neurosci. 2, 63–68 10.1038/35048069 11253360

[B18] Miller J. A. Horvath S. Geschwind D. H. (2010). Divergence of human and mouse brain transcriptome highlights Alzheimer disease pathways. Proc. Natl. Acad. Sci. U.S.A. 107, 12698–12703 10.1073/pnas.0913697107 20616000 PMC2906579

[B19] Nedergaard M. Ransom B. Goldman S. A. (2003). New roles for astrocytes: redefining the functional architecture of the brain. Trends Neurosci. 26, 523–530 10.1016/j.tins.2003.08.008 14522144

[B20] Ng L. Bernard A. Lau C. Overly C. C. Dong H.-W. Kuan C. (2009). An anatomic gene expression atlas of the adult mouse brain. Nat. Neurosci. 12, 356–362 10.1038/nn.2281 19219037

[B21] Ng L. Pathak S. Kuan C. Lau C. Dong H.-W. Sodt A. (2007). Neuroinformatics for genome-wide 3-d gene expression mapping in the mouse brain. IEEE/ACM Trans. Comput. Biol. Bioinform. 4, 382–393 10.1109/tcbb.2007.1035 17666758

[B22] Oberheim N. A. Takano T. Han X. He W. Lin J. H. C. Wang F. (2009). Uniquely hominid features of adult human astrocytes. J. Neurosci. 29, 3276–3287 10.1523/JNEUROSCI.4707-08.2009 19279265 PMC2819812

[B23] Oldham M. C. Konopka G. Iwamoto K. Langfelder P. Kato T. Horvath S. (2008). Functional organization of the transcriptome in human brain. Nat. Neurosci. 11, 1271–1282 10.1038/nn.2207 18849986 PMC2756411

[B24] Pavlidis P. Noble W. S. (2001). Analysis of strain and regional variation in gene expression in mouse brain. Genome Biol. 2, RESEARCH0042 10.1186/gb-2001-2-10-research0042 11597334 PMC57797

[B25] Pearson K. (1901). On lines and planes of closest fit to systems of points in space. Philos. Mag. 2, 559–572

[B26] R Core Team (2012). *R: A Language and Environment for Statistical Computing* Vienna: R Foundation for Statistical Computing.

[B27] Sandberg R. Yasuda R. Pankratz D. G. Carter T. A. Del Rio J. A. Wodicka L. (2000). Regional and strain-specific gene expression mapping in the adult mouse brain. Proc. Natl. Acad. Sci. U.S.A. 97, 11038–11043 10.1073/pnas.97.20.11038 11005875 PMC27144

[B28] Sibille E. Arango V. Joeyen-Waldorf J. Wang Y. Leman S. Surget A. (2008). Large-scale estimates of cellular origins of mRNAs: enhancing the yield of transcriptome analyses. J. Neurosci. Methods 167, 198–206 10.1016/j.jneumeth.2007.08.009 17889939 PMC2262176

[B29] Strand A. D. Aragaki A. K. Baquet Z. C. Hodges A. Cunningham P. Holmans P. (2007). Conservation of regional gene expression in mouse and human brain. PLoS Genet. 3:e59 10.1371/journal.pgen.0030059 17447843 PMC1853119

[B30] Ward J. H. (1963). Hierarchical grouping to optimize an objective function. J. Am. Stat. Assoc. 58, 236 10.1080/01621459.1963.10500845

[B31] Wheeler D. L. Barrett T. Benson D. A. Bryant S. H. Canese K. Chetvernin V. (2007). Database resources of the national center for biotechnology information. Nucleic Acids Res. 35, D5–D12 10.1093/nar/gkl1031 17170002 PMC1781113

[B32] Zapala M. A. Hovatta I. Ellison J. A. Wodicka L. Rio J. A. D. Tennant R. (2005). Adult mouse brain gene expression patterns bear an embryologic imprint. Proc. Natl. Acad. Sci. U.S.A. 102, 10357–10362 10.1073/pnas.0503357102 16002470 PMC1173363

[B33] Zeng H. Shen E. H. Hohmann J. G. Oh S. W. Bernard A. Royall J. J. (2012). Large-scale cellular-resolution gene profiling in human neocortex reveals species-specific molecular signatures. Cell 149, 483–496 10.1016/j.cell.2012.02.052 22500809 PMC3328777

