# Supplementary figures and images for: Neuron-Enriched Gene Expression Patterns are Regionally Anti-Correlated with Oligodendrocyte-Enriched Patterns in the Adult Mouse and Human Brain
Source: Front Neurosci. 2013 Feb 4;7:5. doi: 10.3389/fnins.2013.00005 (PMC3578349; doi:10.3389/fnins.2013.00005)

# H0351.2001 PC 1 gene loadings

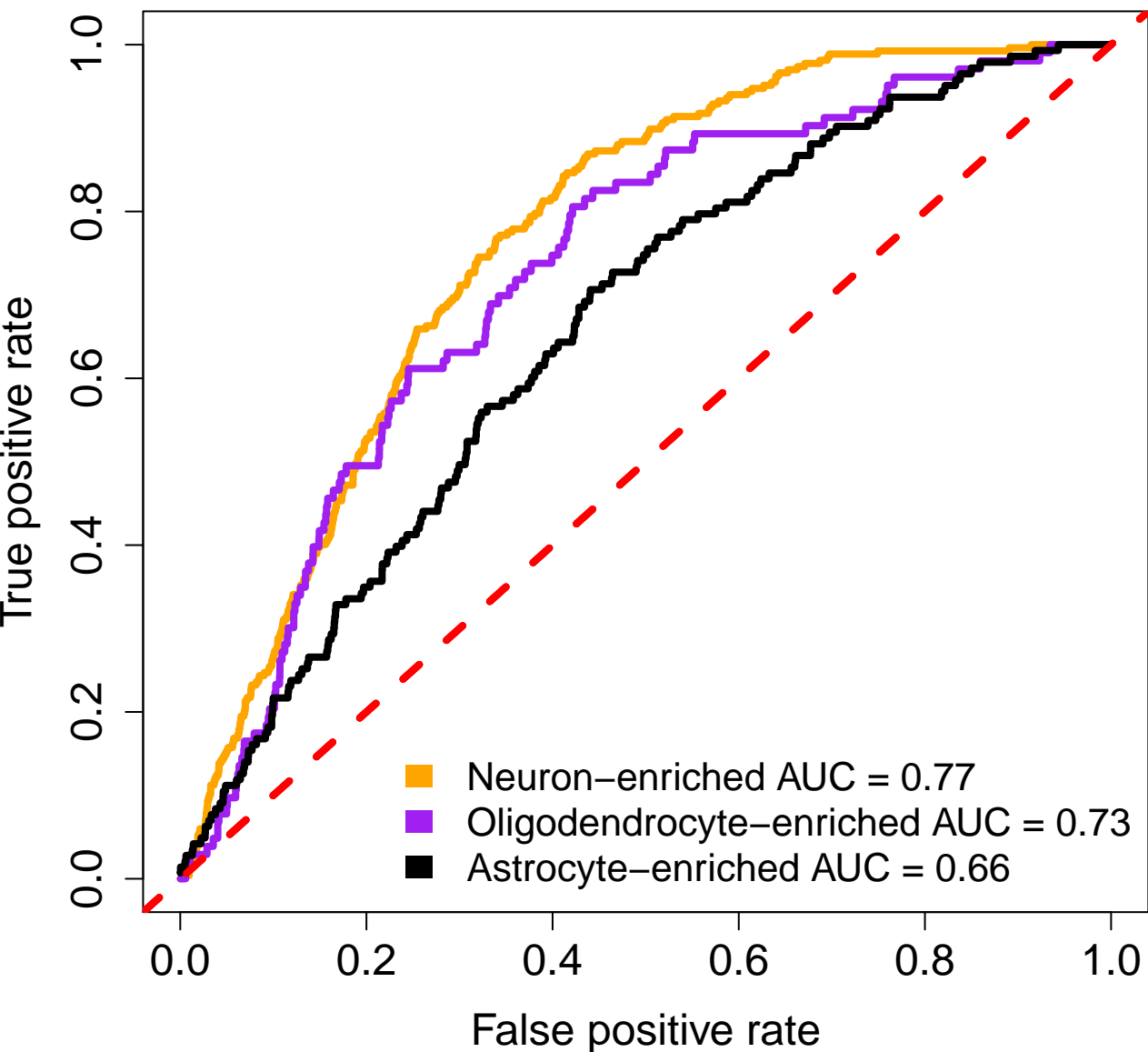

Supplement: Supplementary Figure S1 — H0351.2001 cell type marker enrichment ROC curves. [file figure-s1-roc-pc-ne-oe-astro-H0351.2001.pdf]

# H0351.2002 PC 1 gene loadings

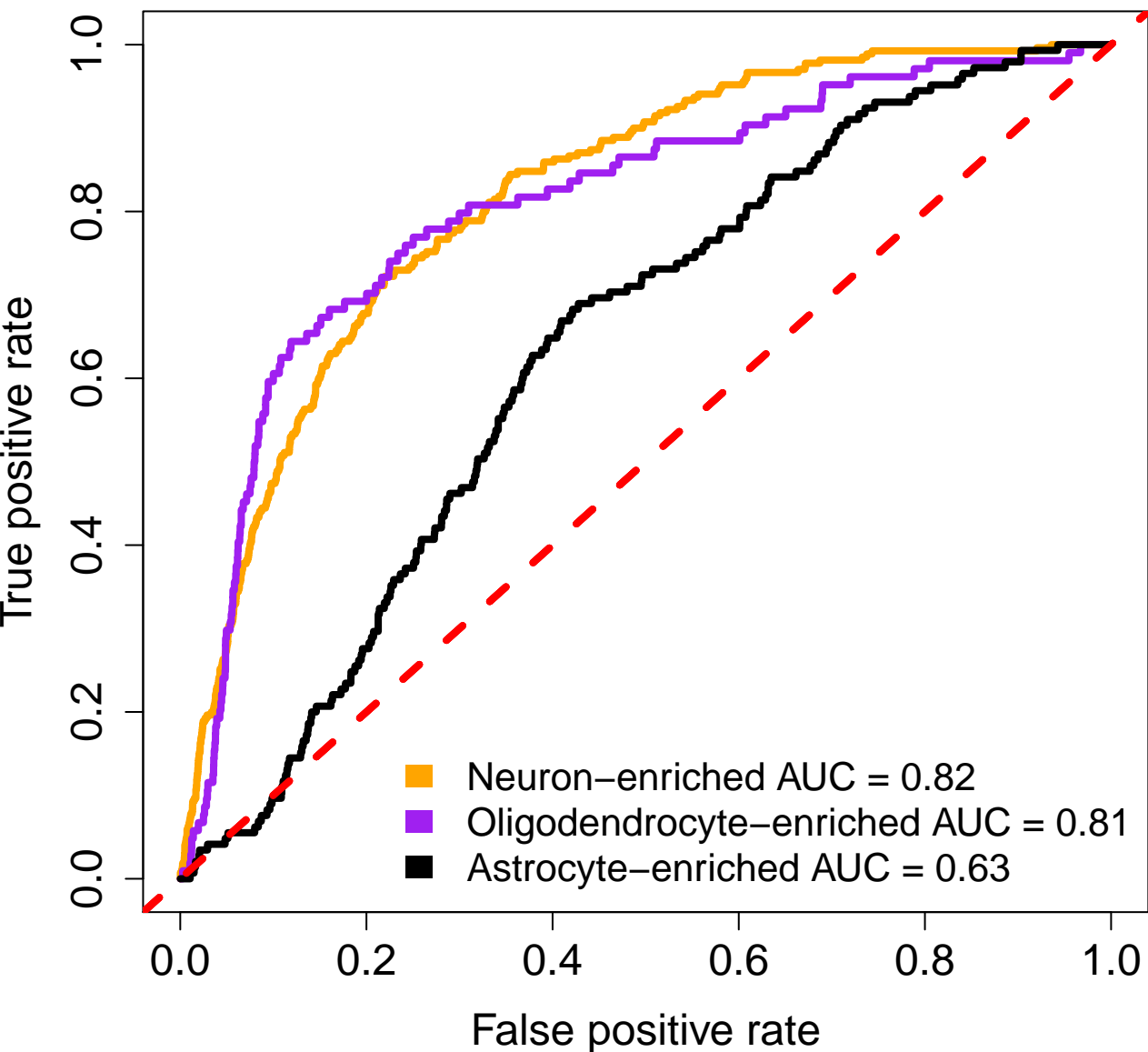

Supplement: Supplementary Figure S2 — H0351.2002 cell type marker enrichment ROC curves. [file figure-s2-roc-pc-ne-oe-astro-H0351.2002.pdf]

## Mouse PC 2 gene loadings

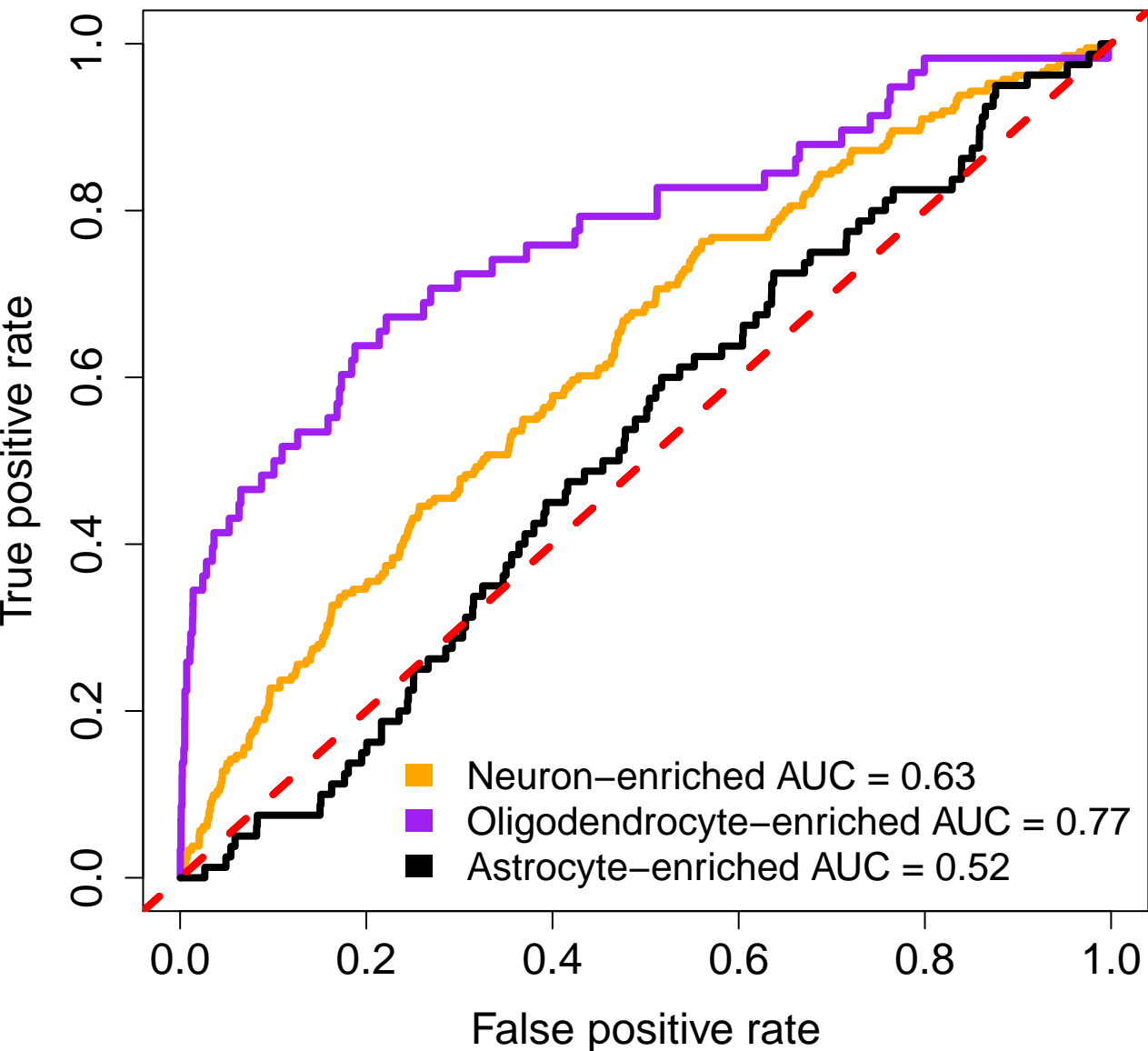

Supplement: Supplementary Figure S3 — Mouse cell type marker enrichment ROC curves. [file figure-s3-roc-mouse-pc-ne-oe-astro.pdf]

# Orthologous gene correlation

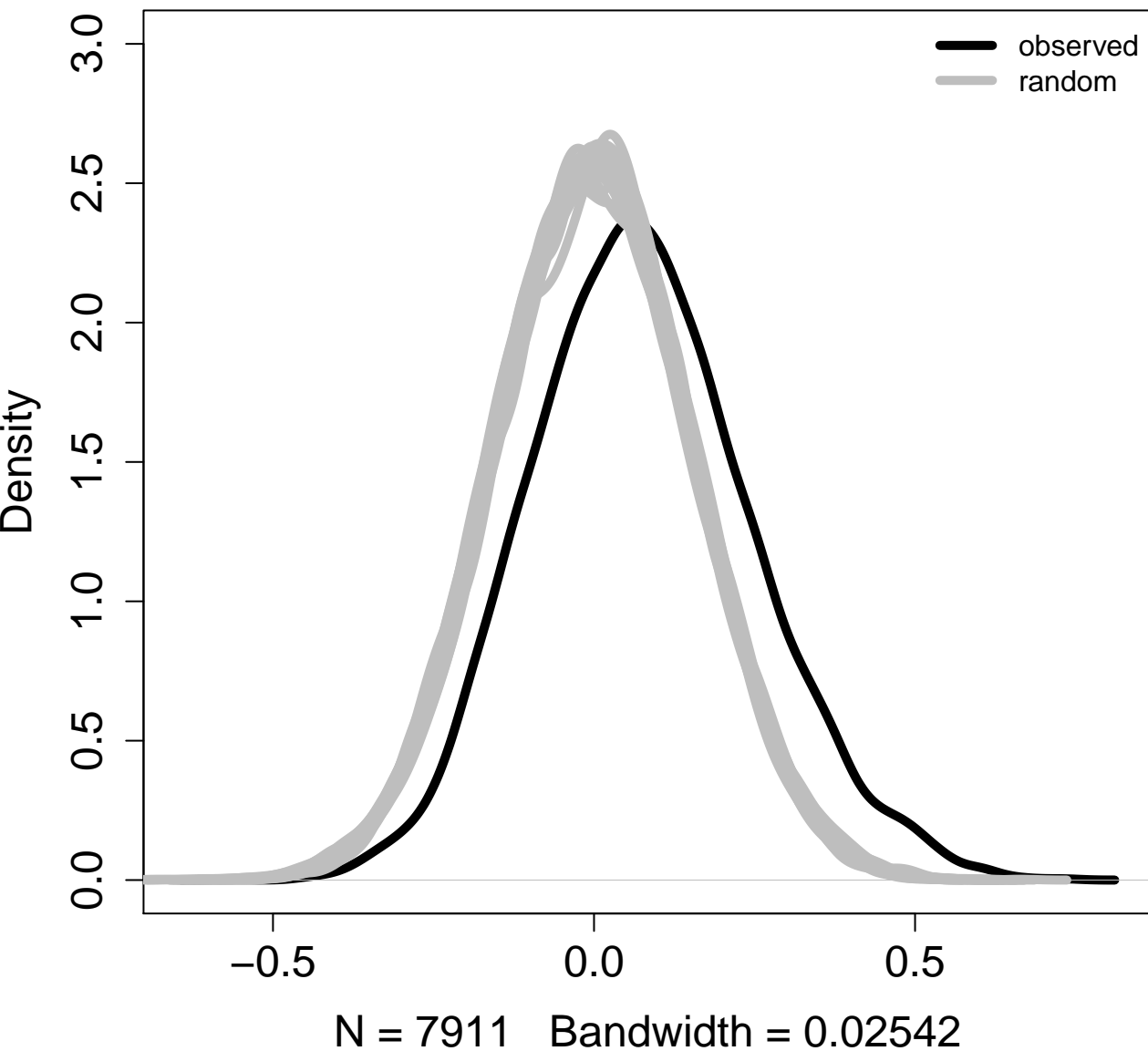

Supplement: Supplementary Figure S4 — Correlation distribution between homologous genes that are expressed. Correlation distribution is skewed toward the positive compared to random where human gene labels were shuffled without replacement. The mean correlation is 0.074. [file figure-s4-hist.mouse.human.cor.diag.skew.gene.permute.pdf]

# Human mouse gene correlation

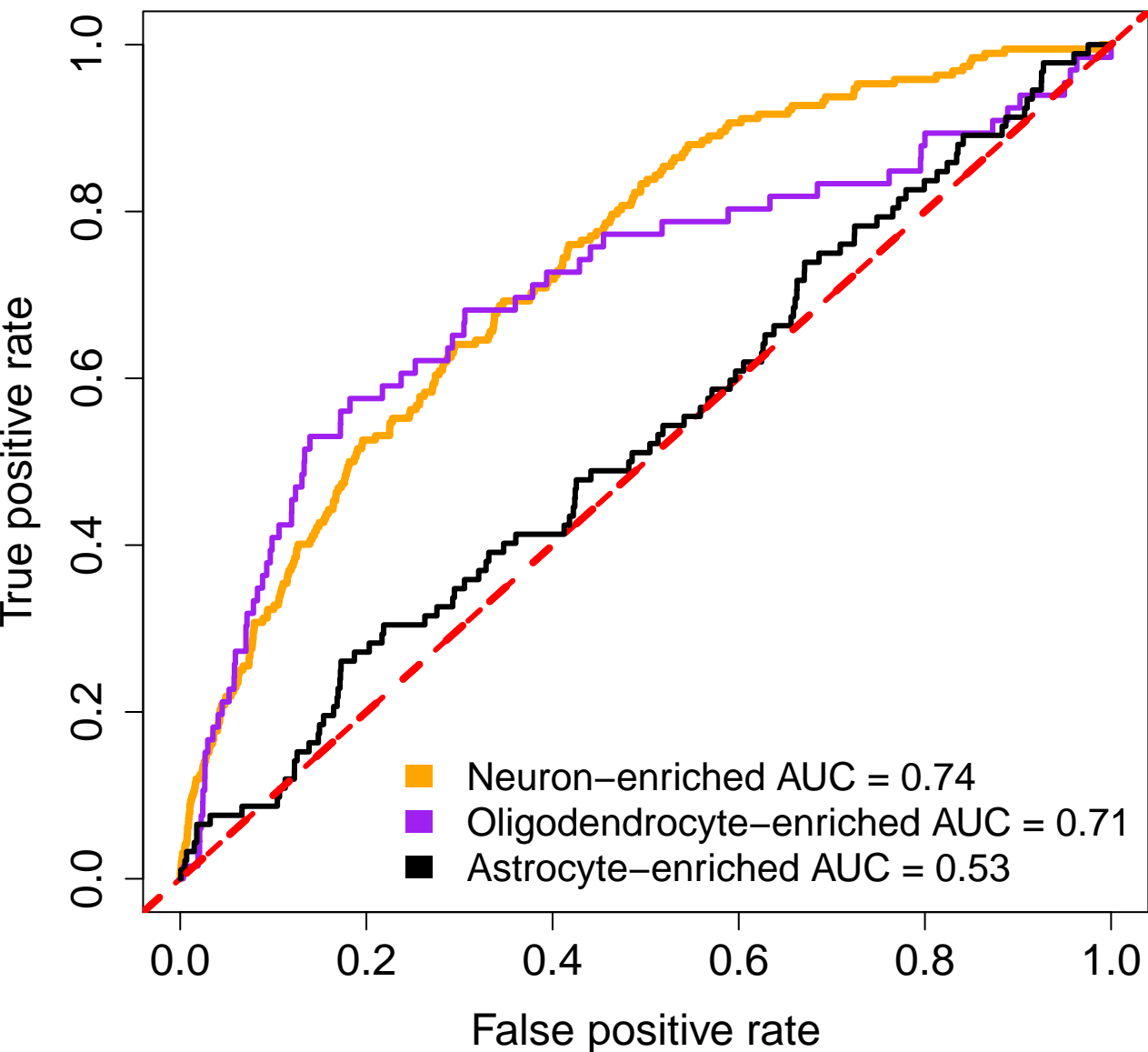

Supplement: Supplementary Figure S5 — Gene-gene correlation cell type marker enrichment ROC curves. Expression data were mean-centered scaled. [file figure-s5-roc-mouse-human-common-cahoy-celltypes.pdf]

**GFAP**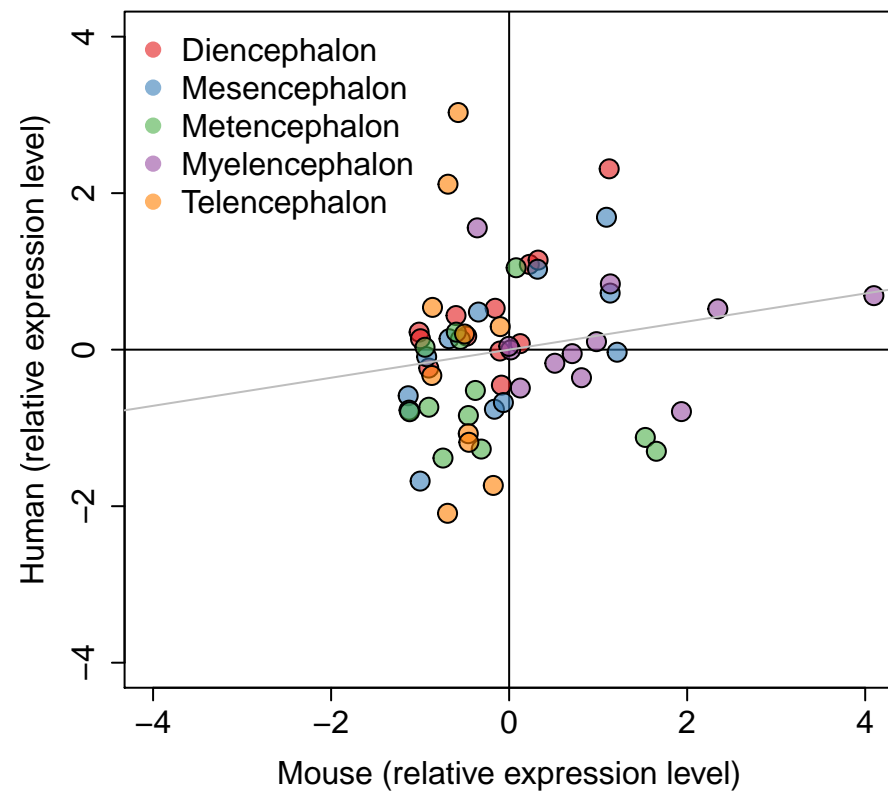**SLC39A12**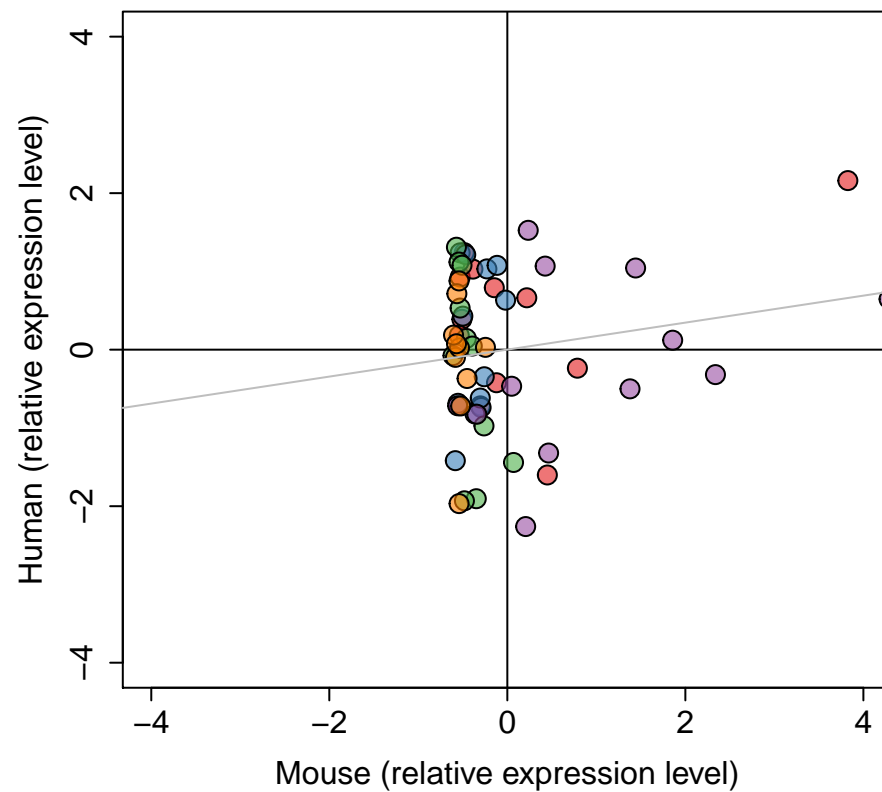**PLA2G7**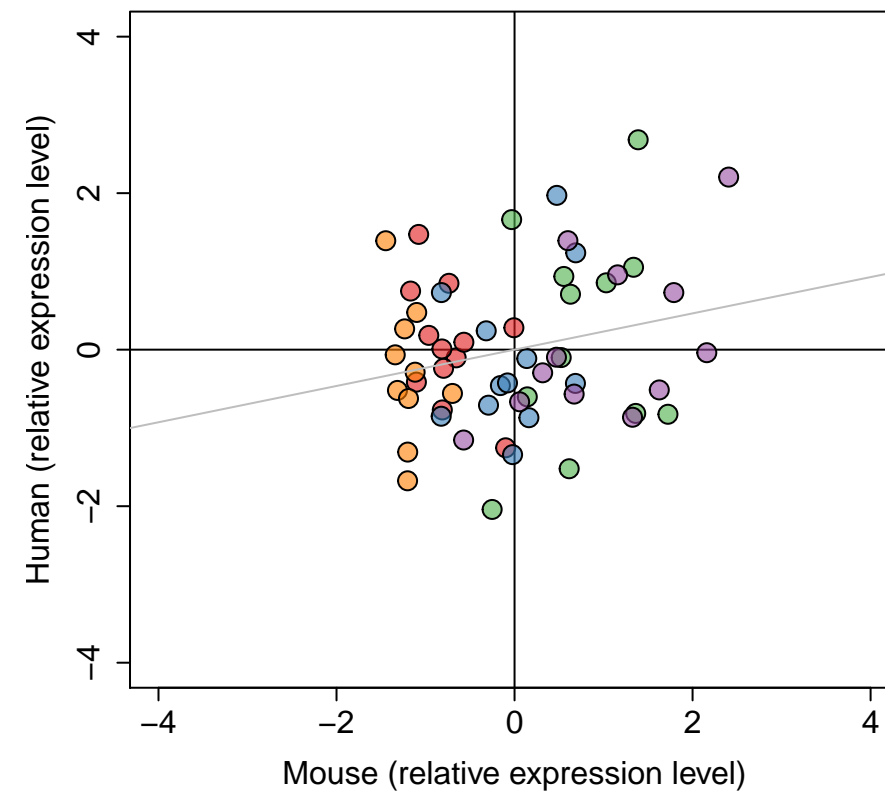**MLC1**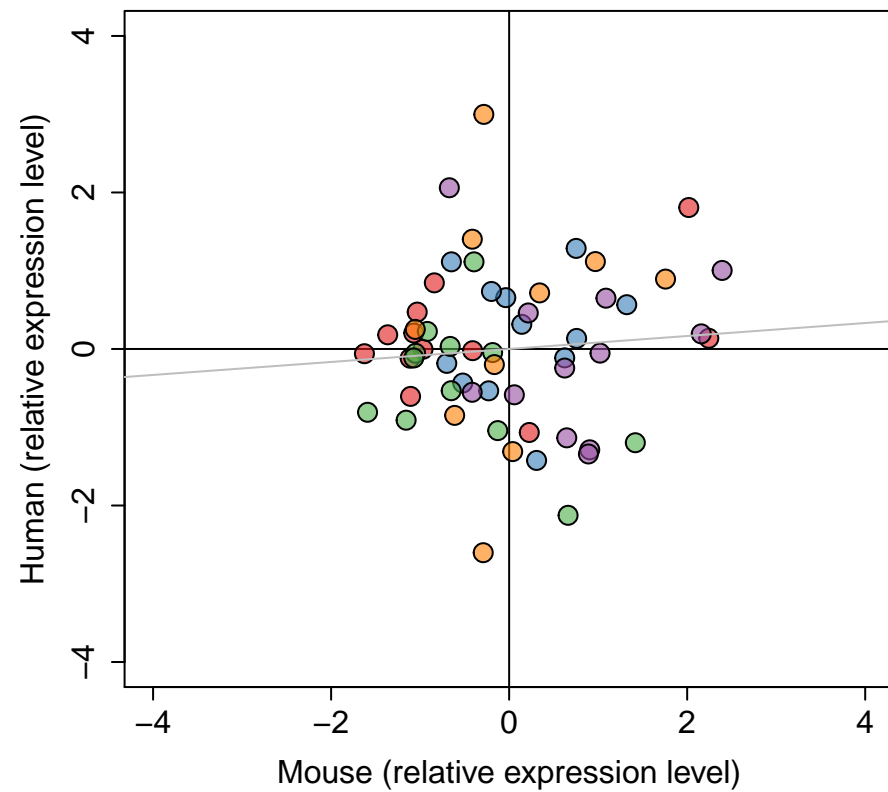**AQP4**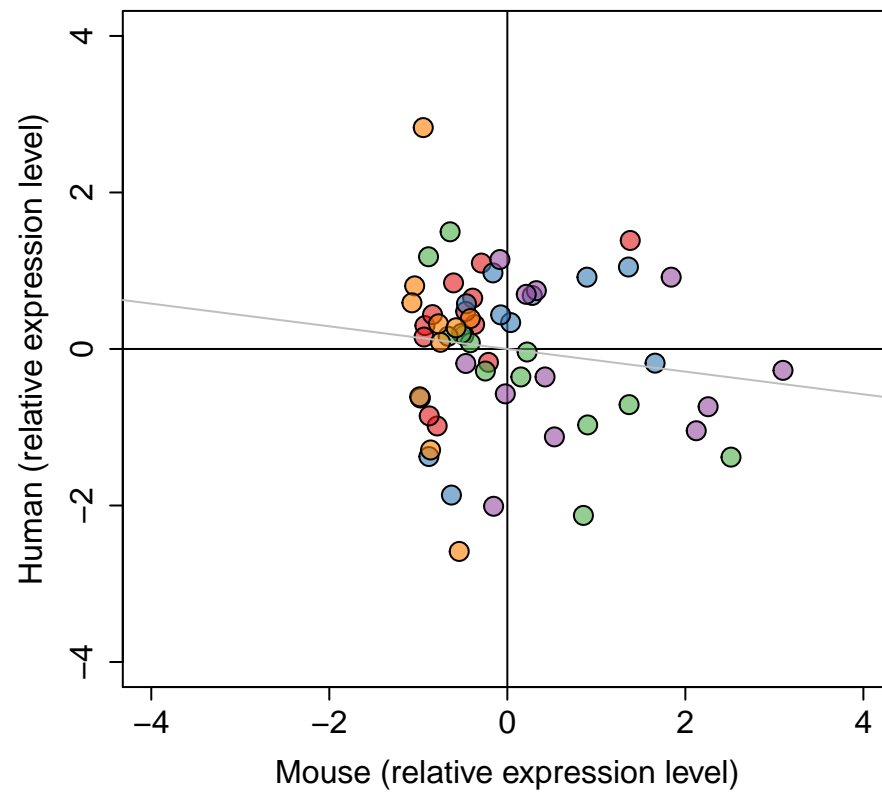**SLC14A1**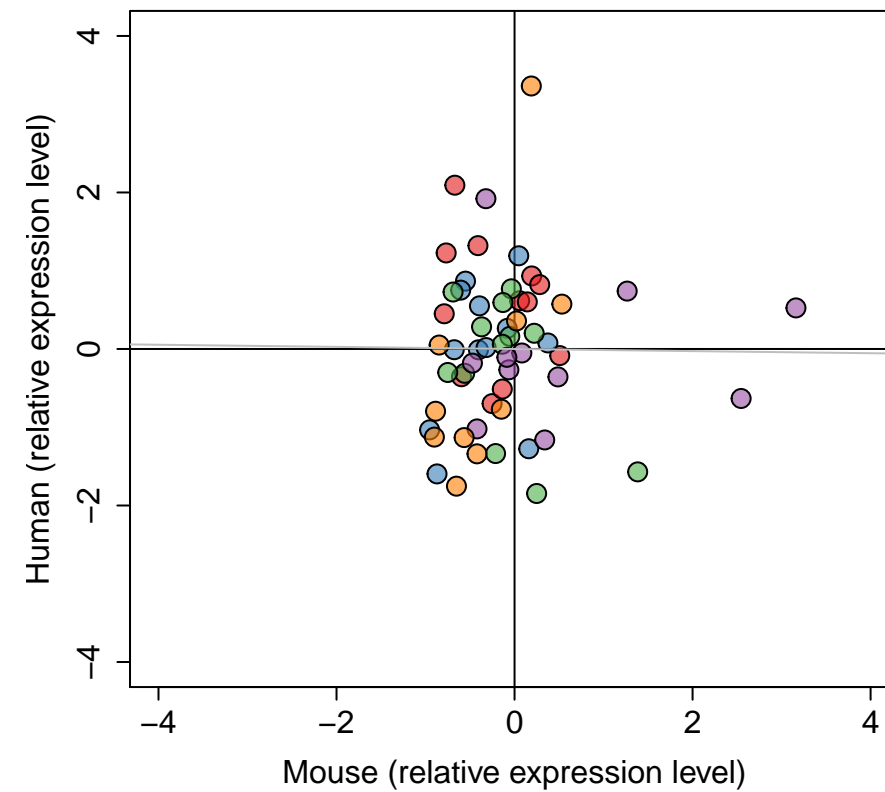

Supplement: Supplementary Figure S6 — Relative expression levels of homologous astrocyte markers across brain regions. [file figure-s6-scatter-mouse-human-cor-expr-astrocyte-genes.pdf]
